# Exotic mangrove *Laguncularia racemosa* litter input accelerates nutrient cycling in mangrove ecosystems

**DOI:** 10.3389/fpls.2024.1463548

**Published:** 2024-10-08

**Authors:** Hongke Li, Chunlian Chen, Jiayi Zhou, He Bai, Shijie Zhang, Qiang Liu

**Affiliations:** Ministry of Education Key Laboratory for Ecology of Tropical Islands, Key Laboratory of Tropical Animal and Plant Ecology of Hainan Province, College of Life Sciences, Hainan Normal University, Haikou, China

**Keywords:** exotic plant, *Laguncularia racemosa*, leaf litter, nutrient cycling, soil microorganisms, microbial decomposers

## Abstract

Exotic plant litter presents different chemical and physical properties relative to native plant litter and alters ecosystem processes and functions that may facilitate exotic plant dispersal. However, these effects are largely unknown, especially within wetland ecosystems. This study examines whether introducing litter from the exotic mangrove *Laguncularia racemosa* could result in (1) accelerated community litter decomposition rates and increased nutrient cycling rates and (2) microbial community structure changes in the invaded areas. A single decomposition experiment using litterbags was conducted to examine the short-term effects of *L. racemosa* litter in the native mangrove forest ecosystem. The soil nutrients and microbial communities of *Rhizophora stylosa*, *L. racemosa*, and mixed forests were also compared to explore the long-term cumulative effects of *L. racemosa* litter in native ecosystems. The results indicated that *L. racemosa* has lower-quality leaf litter than *R. stylosa* and a significantly faster decomposition rate. This may result from changes in the soil microbial community structure caused by *L. racemosa* leaf litter input, which favors the decomposition of its own litter. Both the short-term and cumulative effect experiments demonstrated that *L. racemosa* leaf litter significantly increased the relative abundance of microbes related to litter decomposition, such as Proteobacteria and Bdellovibrionota, and enhanced the alpha diversity of soil fungi, thus creating a microbial environment conducive to *L. racemosa* leaf litter decomposition. Moreover, the accumulation of soil nutrients was lower under *L. racemosa* than under *R. stylosa* over several years. This may be related to the more rapid growth of *L. racemosa*, which causes soil nutrient absorption and storage within the plant tissues, thereby reducing the soil nutrient content. Inputting exotic mangrove *L. racemosa* leaf litter reduced the soil blue carbon content, potentially adversely affecting global climate change. *L. racemosa* may employ a unique strategy to lower soil nutrient levels in native mangroves based on its low-quality leaf litter, thereby weakening the competitive ability of native plants that are intolerant to low-nutrient conditions and enhancing its own competitive advantage to further spread into these areas. In summary, the input of exotic *L. racemosa* leaf litter accelerates nutrient cycling in local mangroves.

## 1 Introduction

Exotic plant invasion is a global phenomenon (Incerti et al., 2018) that threatens the biodiversity and stability of local ecosystems and profoundly impacts their functions and processes (Liao et al., 2008; Meisner et al., 2012; Woodworth et al., 2020). The mangrove *Laguncularia racemosa*, native to La Paz, Mexico (Zhong et al., 2011), is characterized by rapid growth, high reproductive capacity, and adaptability (Zhu et al., 2023). Since its introduction to Hainan, China, in 1999, it has rapidly spread and integrated into local mangrove communities (Liu et al., 2019). Numerous physiological and ecological studies have indicated that *L. racemosa* exhibits higher growth and reproductive abilities compared to native mangrove species in China, suggesting its potential invasiveness (Li et al., 2020; Feng et al., 2022). Research by Lang et al. (2022) also suggested that *L. racemosa* might invade higher latitudes and colder coastal regions of China. Additionally, Zhu et al. (2023) confirmed its invasion potential through field surveys and molecular studies, indicating that it may replace native mangrove species and reduce species diversity within local mangrove forests. Although studies have examined the potential invasiveness of *L. racemosa* from different perspectives, research focusing on associated changes in ecosystem functions remains lacking.

Exotic plants can alter the soil characteristics of invaded areas through their litter inputs, which impact the nutrient cycling within the ecosystem and create positive feedback for the spread of exotic plants (Meisner et al., 2012). Generally, exotic plant litter has higher quality and faster decomposition rates than native plant litter (Prescott and Zukswert, 2016; Woodworth et al., 2020; Hu et al., 2022), and its input often enhances the leaf litter quality in the invaded area, thereby accelerating leaf litter decomposition rates (Ehrenfeld, 2010), increasing soil nutrient contents (Liao et al., 2008), and accelerating nutrient cycling processes (Rothstein et al., 2004). Several studies have shown that these changes may promote fast-growing, nutrient-demanding exotic plants (Meisner et al., 2012) by creating favorable conditions for their spread (Allison and Vitousek, 2004). Additionally, changes in leaf litter quality due to exotic plant litter inputs have been shown to alter microbial communities in the soil, thereby affecting the detrital food web of the local ecosystem (Woodworth et al., 2020) and driving soil nutrient environments toward conditions more conducive to exotic plant growth.

Leaf litter decomposition plays a critical role in the global carbon cycle by regulating organic carbon exchange between the soil and atmosphere (Zhang et al., 2021; Bonanomi et al., 2023). The process of exotic plant invasion is often accompanied by the input of leaf litter with low carbon contents and high nitrogen and phosphorus contents (Kennedy and El-Sabaawi, 2017; Carrasco-Barea et al., 2022), which can impact the soil carbon content and nutrient cycling in the invaded area. Research has shown that the soil under exotic plants has a higher carbon content than soil under native plants (Sanon et al., 2012; Bray et al., 2017); however, studies on the effects of exotic plant litter on soil carbon content are limited. Moreover, although previous studies have primarily focused on how exotic plant invasion affects soil nutrient availability in terrestrial ecosystems, the effects of such invasions on the soil carbon content and nutrient cycling in wetland ecosystems have not been reported. The carbon content of mangrove ecosystems, known as blue carbon ecosystems, is among the highest of ecosystems on Earth (Lu et al., 2024). Since its introduction to China, *L. racemosa* has spread widely across the southeastern coastal provinces of China (Zhong et al., 2011) and invaded local mangrove communities (Liu et al., 2019). However, the impact of its litter input on soil nutrient cycling and blue carbon content in wetland ecosystems remains to be explored.

The impact of leaf litter decomposition on soil nutrients has been studied across various time scales, including the short-term effects of a single litter decomposition event (Meisner et al., 2012; Chen et al., 2020; Zhang et al., 2021) and the long-term effects of continued decomposition over several decades (Lin et al., 2003). However, few studies have integrated both short-term and long-term effects. Since the impacts of leaf litter decomposition on soil nutrients accumulate gradually, short-term experiments cannot provide insights into the effects over longer time scales (Liu and Peng, 2010). Thus, short-term impacts cannot be used to predict long-term effects. Understanding both short-term and long-term cumulative effects is crucial for investigating the impact and mechanisms of leaf litter decomposition on soil nutrient cycling. Therefore, both short-term and long-term effects should be examined to explore how the input of exotic mangrove *L. racemosa* litter affects nutrient cycling in mangrove ecosystems.

Thus, this study aimed to explore the effects of *L. racemosa* leaf litter on nutrient cycling in native mangrove forests from an ecosystem function perspective. We investigated the mangrove forest at Dongzhai Port using the litterbag decomposition method and *in situ* direct sampling to examine the short-term and long-term effects of *L. racemosa* litter input on nutrient cycling in native mangrove ecosystems. We hypothesized that the introduction of litter from the exotic *L. racemosa* could (1) accelerate the decomposition rates of community litter and increase the nutrient cycling rates within the local ecosystem and (2) change the microbial community structure in the invaded areas.

## 2 Materials and methods

### 2.1 Experimental site

The study was conducted at the Dongzhaigang National Nature Reserve, Haikou City, Hainan Province, China (19°51′ to 20°01′N, 110°32′ to 110°37′E). This region features a tropical monsoon maritime climate, with an annual average temperature of 23.3–23.8°C and an annual average rainfall of 1676.4 mm (He et al., 2019). The soil types in the area consist of saline sandy loam or saline marsh soil, with a thickness of approximately 1.0–1.5 m (Cai et al., 2022).

### 2.2 Experimental species

The spread of *L. racemosa* is accompanied by the input of its leaf litter. To better understand the role of *L. racemosa* leaf litter during its dispersal, we selected three types of mangrove forests within the Dongzhai Harbor Nature Reserve: a native mangrove area dominated by *Rhizophora stylosa* (*R. stylosa* forest), a mixed forest of naturally invaded *L. racemosa* and *R. stylosa* (mixed forest), and an experimentally planted mangrove forest dominated by *L. racemosa* (*L. racemosa* forest). The stages represented three processes of *L. racemosa* spread: a native mangrove area where *L. racemosa* has not invaded, a mixed forest during the spread, and a predominantly *L. racemosa* forest after replacement of the native mangrove.

By examining the long-term cumulative effects of *L. racemosa* leaf litter decomposition in the three forests and its short-term effects after a single decomposition event in *R. stylosa* forests, we explored the effects of *L. racemosa* leaf litter input on nutrient cycling, which is a key ecosystem function in native mangrove forests. The *L. racemosa* forests were established in 2013 (20 years of growth). Therefore, this study focused on the cumulative effects of *L. racemosa* litter decomposition over the 20-year period. *Rhizophora stylosa* is an evergreen tree in the Rhizophoraceae family, and it reaches heights of up to 8 m and is highly resistant to wind and wave impacts. It is one of the most representative mangrove species in China (Pan et al., 2018). Field surveys have reported its cooccurrence with the exotic species *L. racemosa* and *Sonneratia apetala* (Liang et al., 2021). We selected *R. stylosa* due to its widespread presence in various native mangrove communities, including those invaded by *L. racemosa* (Liu et al., 2019). *Rhizophora stylosa* covers a significant mangrove area on Hainan Island and accounts for 40% of the total mangrove area (Fang et al., 2022). Moreover, the probability of mixed growth between *L. racemosa* and *R. stylosa* is high, as was observed frequently during field surveys. Thus, the combination was highly representative in our study.

### 2.3 Experimental design

Both the long-term cumulative effects and short-term impacts of *L. racemosa* leaf litter decomposition on native mangrove ecosystems were studied to explore the influence of this species on nutrient cycling and soil microorganisms.

For the long-term cumulative effects study, we examined the impact of inputting *L. racemosa* litter on the long-term accumulation of soil nutrients and microorganisms in native mangrove soils by comparing soil nutrient elements and microorganisms across the three representative mangrove forest types in the study area: *R. stylosa* forest, mixed forest, and *L. racemosa* forest. In June 2023, three plots measuring 10 m × 10 m each were established in the understory of each forest type, with at least 10 m between plots. Within each plot, a five-point sampling method was employed to collect soil samples for nutrient and microorganism analysis. Soil samples from a depth of 0–15 cm were collected using a soil auger for nutrient analysis, which included measurements of organic C, total N (TN), total P (TP), and ammonium N (NH_4_^+^-N). Additionally, 5 g soil samples were collected in cryotubes, stored in liquid N, and subsequently analyzed to determine their bacterial and fungal communities.

For the short-term effects study after a single decomposition experiment, we conducted *in situ* decomposition experiments under the *R. stylosa* forests using litter decomposition bags. Fresh healthy leaves of *L. racemosa* and *R. stylosa* were collected, air-dried, and then placed into bags (1 mm mesh nylon bags at 20 cm × 25 cm) with varying types of litter: *R. stylosa* litter bags, *R. stylosa* and *L. racemosa* mixed litter bags, and *L. racemosa* litter bags. The bags represented scenarios with no *L. racemosa* litter input (R1), mix of *L. racemosa* and *R. stylosa* litter input (R2), and pure *L. racemosa* litter (R3), which replaced the native mangrove litter.

In October 2023, *in situ* decomposition experiments were conducted in three plots in the *R. stylosa* forest to explore the impact of *L. racemosa* leaf inputs on soil nutrients and microorganisms in native mangrove soils. In each plot, 27 bags of each type of litter were placed adjacent to each other, with 9 retrievals per plot and 3 replicates per retrieval, for a total of 243 litter decomposition bags across all plots. Each decomposition bag contained 15 g of air-dried leaves. After removing surface debris, the bags were secured on the soil surface with homemade stakes to prevent tidal displacement, thus ensuring that the bags were closely arranged without overlapping. Decomposition bags were retrieved at 0, 7, 14, 21, 28, 42, 70, 103, and 149 days. The bags were washed and dried to a constant weight, and the remaining mass of the litter was measured to investigate the decomposition rates and remaining percentages for the three types of litter. The chemical properties of the litter collected at 0 and 103 days were analyzed to assess the impact of *L. racemosa* litter input on the quality and nutrient release rate of the native mangrove litter substrate. At 103 days of decomposition, samples from a depth of 0–15 cm beneath the three types of litter bags were collected for nutrient analysis, whereas samples at the surface beneath the bags were collected for microbial analysis. These analyses aimed to investigate the short-term effects of *L. racemosa* litter input on soil nutrients and microorganisms in native mangrove soils. Since the remaining mass at the end of decomposition (149 days) was insufficient for analysis, samples from the 103-day mark were selected for detailed analysis.

### 2.4 Sample handling and measurements

Leaf and soil organic C contents were determined using the potassium dichromate-volumetric method. TN was quantified using the Kjeldahl method, and TP was assessed using the molybdenum antimony anti-colorimetric method. Soil NH_4_^+^-N was quantified using the indophenol blue colorimetric method (Bao, 2000). Leaf lignin contents and cellulose were quantified using a Multiskan™ GO multifunctional microplate reader (Thermo Fisher Scientific, Waltham, MA, USA) (Foster et al., 2010).

The soil bacterial and fungal community compositions were analyzed using the Illumina MiSeq sequencing platform. DNA was extracted from the soil samples using a MagPure Soil DNA LQ Kit (Magan). DNA concentration and purity were assessed using a NanoDrop 2000 spectrophotometer (Thermo Fisher Scientific) and agarose gel electrophoresis, respectively. For the bacterial diversity analysis, genomic DNA served as a template for amplifying the V3–V4 variable region of the 16S rRNA gene (using universal primers 343F and 798R). For the fungal diversity analysis, the ITS1 variable region of the ITS gene was amplified using the primers ITS1F and ITS2. The primer sequences were trimmed using Cutadapt software, and the data were processed using the default parameters of QIIME 2 software (version 2020.11). Representative sequences and amplicon-sequence variant abundance tables were obtained, and the classify-sklearn classifier was employed to annotate representative sequences against the SILVA (version 138; for 16S rRNA) and UNITE (for ITS rRNA) databases. To facilitate downstream diversity and composition analyses, each sample was rarefied to the minimum sequence depth. Species alignment annotations were further analyzed using QIIME 2 software (version 2022.2).

### 2.5 Data processing and analysis

The following formula was used to determine the remaining mass percentage (Hu et al., 2022):


(1) $$\text{Remaining mass percentage}=\frac{M_{t}}{M_{0}}\times 100\%$$

where *M_0_* is the initial litter mass and *M_t_* is the remaining litter mass after time (*t*) of decomposition.

The litter decomposition rate was estimated using the exponential decay model proposed by Olson (1963) and defined as follows:


(2) $$\text{ln}\left(\frac{M_{t}}{M_{0}}\right)=X^{-kt}\text{or y}=ae^{-kt}$$

where *X* is the intercept, *k* is the litter-decomposition constant, y represents *M_t_*/*M_0_*, *a* is the negative natural logarithm of *X*, and *e* is the base natural logarithm.

The formula for determining the mean relative decomposition rate (MRD) was derived from Patil et al. (2020):


(3) $$MRD\left(g\;g^{-1}\text{ y}ear^{-1}\right)=\frac{\ln\left(M_{1}-M_{0}\right)}{t_{1}-t_{0}}$$

where *t_1_*–*t_0_* is the sampling interval in days.

The formulas used to calculate the time required for 50% and 95% litter decomposition (Bockheim et al., 1991; Zhang et al., 2021; Tao et al., 2023) were as follows:


(4) $$t_{50}=\frac{\text{ln}\left(0.5\right)}{\left(-k\right)}$$

(5) $$t_{95}=\frac{\text{ln}\left(0.95\right)}{\left(-k\right)}$$

The collected data were analyzed using IBM SPSS Statistics 22 (IBM Corp., Armonk, NY, USA) and MS Excel (Microsoft Corp., Redmond, WA, USA). Before conducting the statistical analyses, all data were assessed for normality and homogeneity of variance. One-way analysis of variance (ANOVA) or Kruskal–Wallis tests were employed to examine the effects of *L. racemosa* litter on the litter mass, decomposition rate, nutrient residues, and soil nutrients. A p-value < 0.05 was considered statistically significant. Pearson correlation analysis was used to examine the relationship between the average relative decomposition rate of litter and the quality of the litter substrate and determine the correlation between microbial phyla and soil nutrient content. SigmaPlot v15.0 (Grafiti LLC., Palo Alto, CA, USA) was used to fit the litter decomposition rates and their exponential decay models. The litter mass loss at each sampling time point was calculated as the difference between the initial and remaining weights. Bacterial and fungal α-diversity were computed using the stats package in R v3.3.1 (R Foundation for Statistical Computing, Vienna, Austria), alpha diversity was measured using the Shannon index, which considers both the richness and evenness of a sample, and calculated based on a rarefied feature table (QIIME diversity core-metrics-phylogenetic; > 2000 reads). Graphs were generated using Origin 2024 software (OriginLab Inc., Northampton, MA, USA).

## 3 Results

### 3.1 Influence of *L. racemosa* leaf litter input on litter decomposition

#### 3.1.1 Remaining mass percentages and decomposition parameters

**Figure 1:**
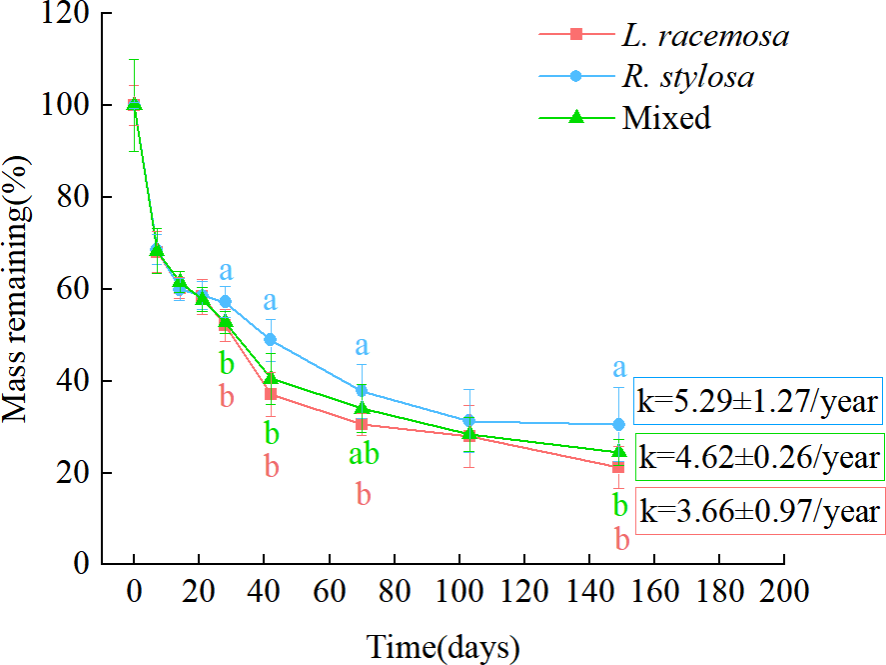
Remaining leaf litter mass percentages from three different types of litter after decomposition in a *R. stylosa* forest. The decomposition constant (*k*) is depicted on the graph as the mean ± standard deviation. Lowercase letters indicate significant differences (p < 0.05) in the remaining mass percentage among the three types of leaf litter.

The remaining mass percentages of leaf litter recovered at each time point in the single decomposition experiment are shown in **Figure 1**. All three litter types exhibited an initial rapid weight loss, followed by a relatively slower decomposition phase. From days 0 to 21 of decomposition, the remaining mass percentages of the three litter types were similar. During the later stages of decomposition (days 28–149, except for day 103), the remaining mass percentages of the *L. racemosa* litter were significantly lower than those of the *R. stylosa* litter. The mixed litter showed consistent patterns with *L. racemosa* except on day 70, when it exhibited significantly lower remaining percentages than *R. stylosa* litter. Based on the remaining mass-percentage data, during the later stages of decomposition, the decomposition rates of the *L. racemosa* and mixed litters were significantly higher than those of the *R. stylosa* litter.

The various decomposition parameters observed via the litter decomposition-bag technique are presented in **Table 1**. An analysis of the annual average leaf litter decomposition rates showed that *L. racemosa* litter decomposed the fastest, *R. stylosa* litter decomposed the slowest, and mixed litter showed an intermediate rate. Curve fitting of the decomposition data for the three leaf litter types revealed a similar pattern of mass loss, with all single litter experiments exhibiting an exponential relationship and presenting R² values from 0.81 to 0.84 and slopes from 3.66 to 5.29. The leaf litter decomposition constant (k) and time required for the litter to lose 50% and 95% of its initial weight suggested that *L. racemosa* litter tended to decompose faster than *R. stylosa* litter, although this trend was not statistically significant.

**Table 1:** Decomposition parameters of leaf litter.

Plant species	Mean relative decomposition rate (g g^−1^ year^−1^)	Decay constant (k) (year^−1^)	Coefficient of determination (R^2^)	Time for 50% decomposition (days)	Time for 95% decomposition (days)
*L. racemose*	5.83 ± 0.14^a^	5.29 ± 1.27^a^	0.85	49.55 ± 10.95^a^	214.14 ± 47.33^a^
*R. stylosa*	5.67 ± 0.18^b^	3.66 ± 0.97^a^	0.81	72.64 ± 20.38^a^	313.94 ± 88.07^a^
Mixture	5.77 ± 0.09^ab^	4.62 ± 0.26^a^	0.84	54.82 ± 3.03^a^	236.99 ± 13.11^a^

Values are the mean ± standard deviation of three replicates (n = 3). Different lowercase letters within each column indicate significant differences (p < 0.05).

#### 3.1.2 Leaf litter substrate quality

Our analysis of the substrate quality of all three types of leaf litter is presented in **Table 2**. *Laguncularia racemosa* leaf litter exhibited significantly lower TN and TP contents and N:P ratios than *R. stylosa* leaf litter but significantly higher C:N, C:P, lignin:N, and lignin:P ratios. Correlation analysis (**Supplementary Table S1**) also indicated that the annual MRD was not significantly correlated with the initial litter quality, suggesting that the substrate quality of *L. racemosa* leaf litter could not explain its faster decomposition.

**Table 2:** Initial chemical characteristics of the three types of litter.

Plant species	C (%)	N (%)	P (%)	Lignin (%)	Cellulose (%)	Lignin: C ratio	Lignin: N ratio	Lignin: P ratio	C: N ratio	C: P ratio	N: P ratio
*L. racemosa*	38.29 ± 2.46^a^	0.32 ± 0.04^c^	0.06 ± 0.01^b^	10.21 ± 0.7^ab^	0.11 ± 0.05^ab^	0.27 ± 0.01^a^	32.32 ± 3.16^a^	163.79 ± 12.91^a^	120.9 ± 8.14^a^	613.66 ± 39.55^a^	5.10 ± 0.52^b^
*R. stylosa*	39.26 ± 3.03^a^	0.48 ± 0.03^a^	0.07 ± 0.01^a^	9.81 ± 1.25^b^	0.14 ± 0.07^a^	0.25 ± 0.04^a^	20.49 ± 3.28^c^	138.53 ± 26.89^b^	81.49 ± 4.72^c^	550.38 ± 59.69^b^	6.75 ± 0.58^a^
Mixture	38.86 ± 2.85^a^	0.42 ± 0.03^b^	0.07 ± 0.01^ab^	10.77 ± 0.65^a^	0.08 ± 0.02^b^	0.28 ± 0.02^a^	25.75 ± 2.06^b^	155.13 ± 13.79^ab^	92.70 ± 5.26^b^	562.20 ± 76.89^ab^	6.06 ± 0.76^ab^

Values are the mean ± standard deviation of three replicates (n = 3). Different lowercase letters within each column indicate significant differences (p < 0.05).

Basal chemical quality analyses of the litter indicated that after *L. racemosa* had begun to spread and replace *R. stylosa* (with all forest floor leaf litter being produced by *L. racemosa*), the quality of the forest floor leaf litter was significantly lower compared to that prior to the spread of *L. racemosa* into the area (i.e., no input of *L. racemosa* leaf litter). The findings suggest that adding *L. racemosa* litter led to a decline in the quality of native mangrove litter.

### 3.2 Impact of *L. racemosa* leaf litter input on soil nutrient cycling

#### 3.2.1 Long-term cumulative effects

**Figure 2:**
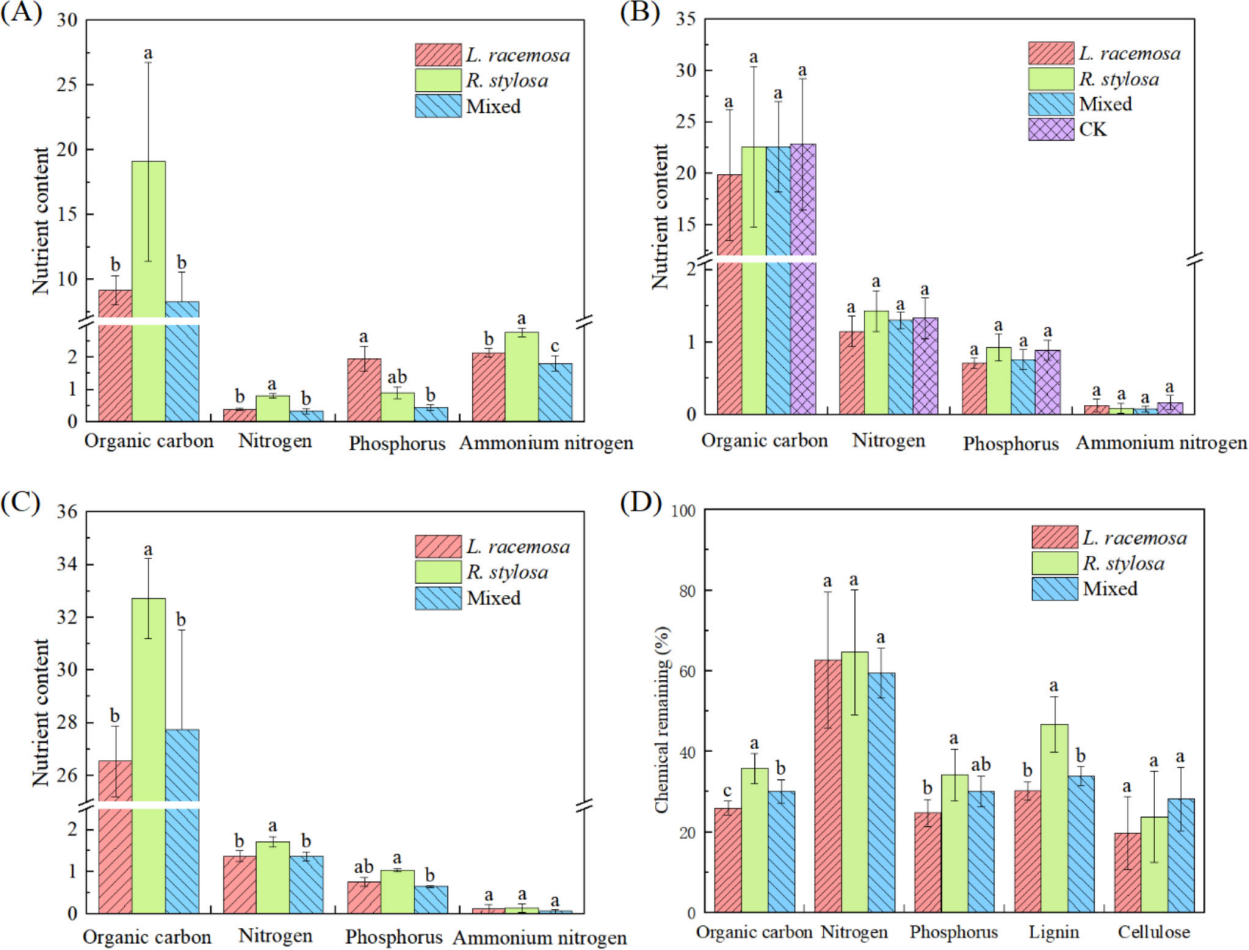
Elemental soil nutrient contents of the three vegetation types. **(A)** Soil element content beneath litter bags after decomposition, total contents **(B)**, and content at the R1 sites **(C)** in grams per kilogram (g/kg) except for ammonium nitrogen, which was measured in milligrams per kilogram (mg/kg). In **(B)** ‘CK’ denotes the control soil site beneath *R. stylosa*, which was characterized by the absence of litter cover and decomposition. **(D)** Nutrient residual rates after litter decomposition. Different lowercase letters indicate significant differences at p < 0.05.

The nutrient content of the soil under all three forest types was determined to investigate the long-term impact of *L. racemosa* leaf litter input on soil nutrients in native mangrove forests. **Figure 2A** illustrates the nutrient content of the soil in all three forest types. The soil organic C, TN, and NH_4_^+^-N contents were significantly higher in the *R. stylosa* forests than the *L. racemosa* forests. Specifically, the organic C and TN contents in the *R. stylosa* forests were more than double those of the *L. racemosa* forests. *R. stylosa* forests, mixed-species forests, and *L. racemosa* forests were used to represent the three stages of *L. racemosa* invasion.

#### 3.2.2 Short-term impacts of the single decomposition experiment

Comprehensive analysis of our data from all three sample sites beneath the *R. stylosa* forests (**Figure 2B**) revealed unexpected results. The nutrient element contents in the soil did not significantly differ (p > 0.05) for the three types of decomposed leaf litter. Furthermore, the nutrient contents in the soil after leaf litter decomposition did not differ significantly from those in the soil without leaf litter decomposition. Although not statistically significant, we did observe that the soil organic C, TN, and TP contents tended to be lower following the single decomposition of *L. racemosa* leaf litter than after the single decomposition of *R. stylosa* litter, with mixed litter showing an intermediate value.

However, we observed significant effects on decomposition at site R1, which was beneath the *R. stylosa* forest (**Figure 2C**). The soil organic C and TN contents at this site were significantly lower after *L. racemosa* and mixed leaf litter decomposition than after *R. stylosa* leaf litter decomposition.

Measuring the residual nutrient rates after short-term decomposition of the three types of leaf litter (**Figure 2D**) revealed that after 103 days of field decomposition, *R. stylosa* leaf litter exhibited the highest residual rates of organic C, P, and lignin (approximately 35, 35, and 45%, respectively), *L. racemosa* showed the lowest residual rates, and mixed leaf litter showed intermediate values. Notably, lignin, with its complex structure, is recalcitrant to decomposition. The significantly lower levels of residual lignin in *L. racemosa* leaf litter compared to *R. stylosa* leaf litter indicated that *L. racemosa* leaves decomposed more readily than *R. stylosa* leaves. Additionally, *L. racemosa* exhibited the lowest residual rates of organic C and P.

### 3.3 Impacts of *L. racemosa* leaf litter input on soil microorganisms

#### 3.3.1 Alpha diversity of microbial communities

**Figure 3:**
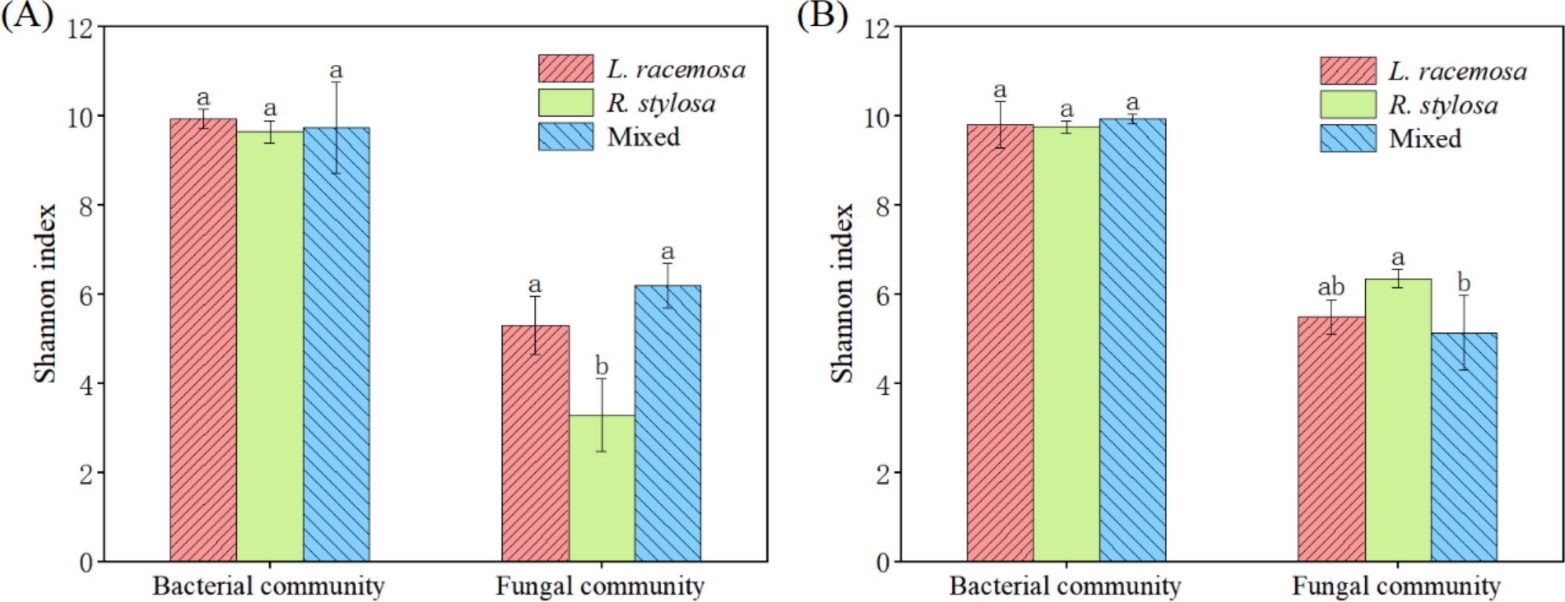
Alpha (α)-diversity indices. **(A)** Understory soil samples from the three sites. **(B)** Soil beneath the litter after decomposition. Bacterial and fungal community α-diversity indices across the three sites, with different lowercase letters indicating significant differences among treatments (p < 0.05).

The bacterial community α-diversity (Shannon index) did not significantly differ among the three forest types in the cumulative effect experiments. However, the Shannon index for fungal communities in the soil under *L. racemosa* and mixed forests was significantly higher than that under *R. stylosa* (**Figure 3A**). Moreover, the bacterial α-diversity in the soil beneath the three types of leaf litter did not significantly differ after decomposition in the single decomposition experiment (**Figure 3B**). The Shannon index for soil fungi after *R. stylosa* leaf decomposition was significantly higher than that after mixed leaf decomposition, which was possibly due to the mixed effect of leaf litter reducing the α-diversity of soil fungi in the short term. However, the long-term cumulative effects of *L. racemosa* spreading into the area will likely reverse this trend and increase the soil fungal α-diversity.

#### 3.3.2 Effects on microbial communities over short and long terms

**Figure 4:**
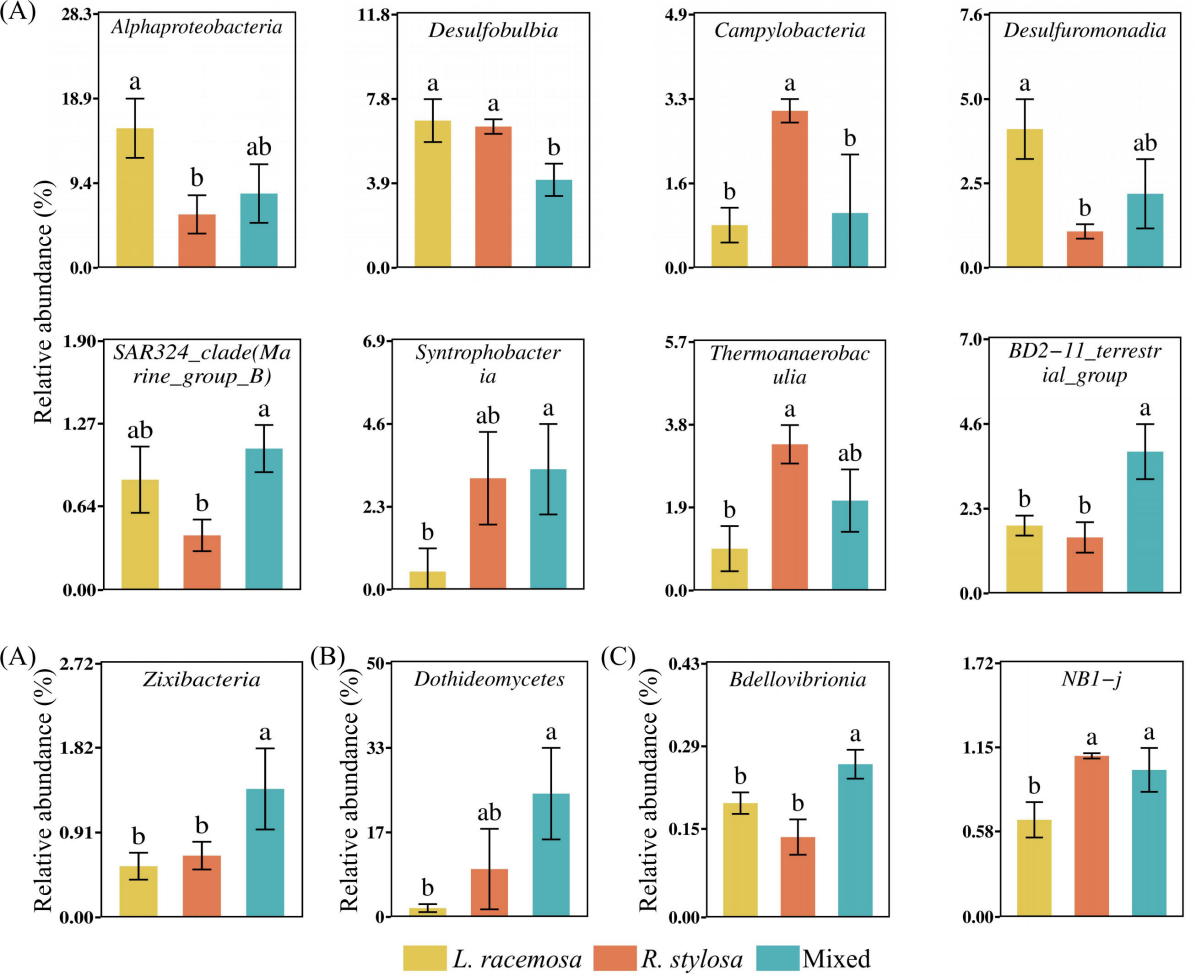
Differential bacterial and fungal class abundance. **(A)** Differences in bacterial **(B)** and fungal classes in the soil beneath the litter across all three sites. **(C)** Differential bacterial classes in the soil beneath the litter after decomposition. Different lowercase letters indicate significant differences among treatments (p < 0.05).

An analysis of the long-term cumulative effects on the bacterial communities in the soil under the three mangrove forest types revealed that 19 bacterial phyla had a relative abundance > 1% (defined as at least one treatment showing an average relative abundance > 1%). These phyla collectively represented over 96% of the total bacteria. Proteobacteria, Desulfobacterota, and Bacteroidetes were the dominant phyla in all three mangrove types (**Supplementary Figure S2A**). Among them, Proteobacteria was significantly more abundant in *L. racemosa* forests than in *R. stylosa* forests, with mixed forests exhibiting intermediate levels. Bdellovibrionota was significantly more abundant in *L. racemosa* forests than in *R. stylosa* forests. At the class level, differential analysis of the top 30 most abundant bacterial classes (**Figure 4A**) revealed that Alphaproteobacteria was significantly more abundant in *L. racemosa* forests than in the mixed and *R. stylosa* forests.

**Figure 5:**
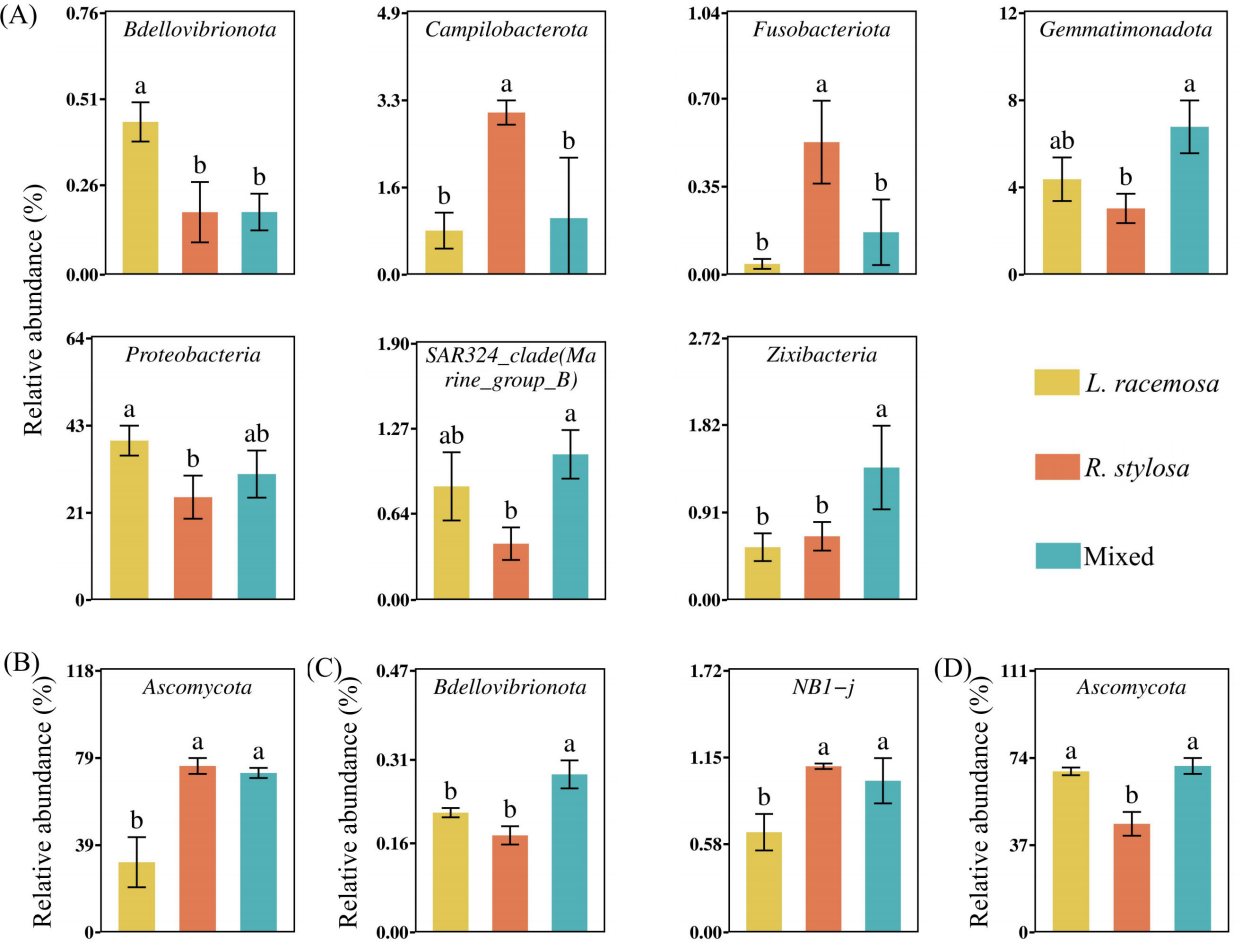
Phylum abundance. **(A)** Differential bacterial **(B)** and fungal phyla in the soil beneath litter of all three forest types. **(C)** Differential bacterial **(D)** and fungal phyla in the soil beneath litter after decomposition. Different lowercase letters indicate significant differences among treatments (p < 0.05).

Regarding the fungal soil communities in the three mangrove forest types, four fungal phyla had a relative abundance > 1%. Ascomycota represented the dominant phylum common in all three mangrove types (**Supplementary Figure S2B**). Differential analysis of the 30 most abundant fungal phyla in the soil of all three mangrove forest types (**Figure 5B**) revealed that Ascomycota, known for its activity in the early decomposition stages, exhibited significantly higher relative abundance in *R. stylosa* and mixed forests than in *L. racemosa* forests. At the class level, Dothideomycetes was significantly more abundant in mixed and *R. stylosa* forests than in *L. racemosa* forests (**Figure 4B**).

Correlation analysis of the microbial phyla (top 30 bacterial and fungal taxa based on relative abundance) and soil nutrient content in the three forest types (**Supplementary Table S2**, showing only microorganisms with significant correlations) revealed significant correlations between the microbial communities and soil nutrient contents. Furthermore, the results of the correlation and relative abundance analyses of differential microorganisms among the three forest types (**Figure 5**) were consistent with the results on the soil nutrient contents in the forests (**Figure 2A**).

An analysis of the short-term effects of the single decomposition litter experiment on the bacterial communities in the soil revealed 14 bacterial phyla with a relative abundance > 1%. These phyla collectively accounted for over 95% of the total bacterial population. Proteobacteria, Desulfobacterota, and Bacteroidota were the predominant phyla in the soil after litter decomposition (**Supplementary Figure S2C**). Differential analysis of the 30 most abundant bacterial phyla in the soil beneath the decomposed litter (**Figure 5C**) revealed significant differences in NB1-j and Bdellovibrionota across all three treatments. Bdellovibrionota, which aids in cellulose decomposition, was significantly more abundant in soils following *L. racemosa* litter decomposition than after *R. stylosa* litter decomposition. At the class level, differential analysis indicated that NB1-j was significantly less abundant in soils after *L. racemosa* litter decomposition than after *R. stylosa* and mixed litter decomposition.

The fungal community composition in the soil beneath the single decomposition experiment is illustrated in **Supplementary Figure S2D**. Differential analysis of the 30 most abundant fungal phyla across all three treatments (**Figure 5D**) revealed that Ascomycota, known for its abundant cellulase genes, was significantly more abundant in soils following *L. racemosa* and mixed litter decomposition than after *R. stylosa* litter decomposition. Moreover, fungal classes did not significantly differ across the three treatments. However, the number of bacterial and fungal species showing significant differences in the soils after the single decomposition experiments was lower than that observed after the cumulative effects multi-year decomposition experiments, indicating that the short-term impacts from small amounts of litter were relatively minor. The cumulative effects of multi-year *L. racemosa* litter decomposition shifted the bacterial and fungal communities in native mangrove soils toward faster decomposition.

Correlation analysis of the microbial phyla (the top 30 bacterial and fungal taxa by relative abundance) in the three soils after a single decomposition experiment revealed that certain soil microbial taxa associated with the decomposition of each leaf litter were significantly correlated with the MRD and soil nutrient content (**Supplementary Table S3**, listing only microorganisms with significant correlations). However, the microbial taxa exhibiting significant correlations in the soil after the single decomposition experiment were not entirely consistent with those exhibiting significant correlations in the understory of the three sites.

## 4 Discussion

### 4.1 Changes in litter decomposition rates in native mangrove forests due to *L. racemosa* leaf litter input

Many studies have shown that the input of exotic plant litter can accelerate the decomposition rate of litter in invaded areas (Liao et al., 2008; Poulette and Arthur, 2012; Chen et al., 2013). In our study, although the litter quality of *L. racemosa* was lower than that of *R. stylosa*, its litter input reduced the litter quality in the invaded sites, which led to accelerated decomposition rates (**Table 1**) and increased nutrient release rates (**Figure 2D**). This finding is consistent with the results of previous reports and supports our first hypothesis that the input of *L. racemosa* litter will accelerate the decomposition rate and nutrient cycling of invaded areas.

In terrestrial ecosystems, the litter quality of exotic plants is generally higher than that of native plants and thus enhances the local litter quality (Woodworth et al., 2020; Patil et al., 2020; Meisner et al., 2012; Zhang et al., 2021). Litter quality is a crucial factor affecting decomposition (Gao et al., 2016; Cheng et al., 2024), and higher-quality litter has been reported to decompose more easily than lower-quality litter (Kennedy and El-Sabaawi, 2017; Carrasco-Barea et al., 2022). However, whether this dynamic occurs in wetland ecosystems, particularly mangrove ecosystems, has not been previously clarified. Our findings on the exotic mangrove *L. racemosa* were inconsistent with those of previous studies. The litter of *L. racemosa* has higher C:N and C:P ratios than the litter of the native mangrove species *R. stylosa*, indicating that it has lower-quality litter. However, the low litter quality of *L. racemosa* accelerated its decomposition in the invaded area (**Table 1**), which is contrary to previous findings. Li et al. (2020) also highlighted the low-quality characteristics of *L. racemosa* litter. Under similar climatic conditions, litter decomposition is regulated by litter quality and decomposer communities (Patil et al., 2020; Zhang et al., 2021; Cheng et al., 2024). Thus, we sought to determine the factors underlying the faster decomposition rate of the lower-quality *L. racemosa* litter. An analysis of the soil microbial community structure revealed that the input of *L. racemosa* litter significantly increased the relative abundance of microbes related to litter decomposition, such as Proteobacteria and Bdellovibrionota, and enhanced the alpha diversity of soil fungi. Thus, a microbial environment conducive to the decomposition of *L. racemosa* leaf litter was established. This issue will be further discussed in the following section.

### 4.2 Alterations in soil microbial communities in native mangrove forests due to *L. racemosa* leaf litter input

We found that the input of *L. racemosa* leaf litter altered the soil microbial community in native mangroves and stimulated the growth of certain dominant bacteria and fungi associated with litter decomposition, such as Proteobacteria, Bdellovibrionota, and Ascomycota. These microbial groups play a crucial role in accelerating leaf decomposition (Gladkov et al., 2022; Andronov et al., 2022; Kimeklis et al., 2023) and presented a higher relative abundance after the input of *L. racemosa* litter; thus, they accelerated the decomposition of the *L. racemosa* litter. The long-term cumulative effects indicated that *L. racemosa* litter decomposition also increased the soil fungal α-diversity. Higher microbial diversity supports elevated levels of ecosystem functions, which further facilitate the decomposition of *L. racemosa* litter. Correlation analyses further revealed that certain soil bacteria and fungi were significantly related to the MRD and nutrient content of the soil after litter decomposition (**Supplementary Tables S2**, **S3**), indicating a correlation between the faster decomposition rates of *L. racemosa* leaf litter and the structure of the soil decomposer community. This supports our hypothesis that the input of *L. racemosa* litter alters the microbial community structure in the invaded area. Additionally, these results suggest that a key reason for the faster decomposition rate of *L. racemosa* litter compared to *R. stylosa* litter may be changes in the soil microbial community structure caused by the input of *L. racemosa* litter. One of the ecological impacts of exotic plant dispersal is the alteration of soil microbial communities, which affects the diversity, abundance, and functionality of microbes, thereby leading to changes in litter decomposition rates and soil nutrient availability (Torres et al., 2021). Specific types of litter have distinct morphological and chemical characteristics that can act as ecological filters that select for or exclude certain microbial groups from the shared soil pool. Variations in the chemical exudates from litter can also directly influence soil microbial communities (Waldrop and Firestone, 2004). Therefore, the input of litter from exotic plants with different morphological and chemical traits leads to alterations in the local soil microbial community composition. Microbial communities with higher abundance and diversity often support higher levels of ecosystem functions, such as biomass decomposition, by facilitating interactions and resource allocation among microbial species (Zhang et al., 2023), thereby accelerating the decomposition process.

A meta-analysis of the effects of invasive plants on soil microbial communities revealed that exotic plants can enhance soil nutrient cycling by altering soil microbes, thus promoting invasion success (Hou et al., 2022). Our study revealed that after the input of *L. racemosa* leaf litter, the α-diversity of soil fungi increased. Changes in the relative abundance of dominant bacteria and fungi associated with leaf litter decomposition led to increased decomposition rates and nutrient release from native mangrove leaf litter. These findings are consistent with those of previous studies on invasive plants (Arthur et al., 2012; Yan et al., 2018; Zhang et al., 2023) and suggest that *L. racemosa* shares similar characteristics with invasive species in terms of modifying soil microbes to enhance soil nutrient cycling. This finding indicates the potential invasiveness of *L. racemosa*.

### 4.3 Decreased soil nutrient contents in native mangrove forests due to *L. racemosa* leaf litter input

Research on the impact of exotic plants on soil nutrient cycling is important for evaluating the effects of these plants (Ehrenfeld, 2003). Exotic plants often have higher leaf litter quality and decomposition rates, leading to increased nutrient content in the soil through leaf decomposition, which alters ecosystem nutrient cycling and promotes exotic plant adaptation and growth (Meisner et al., 2012; Patil et al., 2020; Rothstein et al., 2004). However, our experiments showed that the short-term and long-term inputs of *L. racemosa* leaf litter resulted in decreased levels of carbon, nitrogen, and ammonium nitrogen in the soil of the invaded native mangrove forests, which is inconsistent with the increase in nutrients typically observed in studies of exotic plant litter (Sanon et al., 2012; Bray et al., 2017).

An area of research is why *L. racemosa* exhibits rapid litter decomposition but causes lower soil nutrient contents. Although the leaf litter of *L. racemosa* decomposes quickly, it has low quality and nutrient contents. However, *L. racemosa* is a tall fast-growing species, suggesting that its rapid growth requires substantial nutrients. Thus, the high amount of nutrients released from the decomposing leaf litter may be absorbed by *L. racemosa* plants to support their growth and development. This hypothesis was validated by Zhu et al. (2023), who performed genomic analyses of the leaves of *L. racemosa* from Dongzhaigang (Hainan Province) and discovered convergent evolution in genes responsible for nutrient absorption, which enhanced N and P uptake and transport capacity. The results of Liu et al. (2020) further supported the hypothesis that the introduced mangrove *L. racemosa* predominantly absorbs and enriches nutrients in plant tissues. They observed significantly higher richness in rhizospheric and endophytic diazotrophic communities as well as rhizospheric nutrient components (such as TN) in *L. racemosa* mangroves than in native mangroves, thus providing evidence based on the rhizospheric microbiota and nutrition. In our study, we also discovered that Alphaproteobacteria, which facilitates N fixation in plants, was significantly more abundant in *L. racemosa* forests than in mixed and *R. stylosa* forests, thus providing further support for our hypothesis. Therefore, the rapid decomposition of *L. racemosa* litter and lower soil nutrient contents might be attributed to the retention of nutrient elements in *L. racemosa* plants to facilitate their own rapid growth and development. In summary, inputting exotic mangrove *L. racemosa* litter accelerated nutrient cycling within mangrove ecosystems. The rapid decomposition of *L. racemosa* litter and the lower soil nutrient contents could reflect the absorption and retention of released nutrients to promote their rapid growth and development. Ecosystem nutrient cycling is a continuous process involving leaf litter decomposition, soil nutrient increase, plant growth, and subsequent leaf litter decomposition. The soil nutrient contents depend not only on the amount of nutrients released during leaf litter decomposition but also on the amount of nutrients absorbed and fixed within the plant biomass, thus reflecting a balance between nutrient inputs and outputs. Due to the rapid growth and high biomass of exotic plants, more nutrients are sequestered in their larger structures, leading to reduced nutrient levels in the soil. Compared to native plants, these larger exotic plants can gain a competitive advantage based on their enhanced absorption of sunlight and uptake of soil nutrients, which further facilitate their spread into new areas.

Many studies have assumed that the impact of exotic plants on soil nutrient cycling enhances the competitive advantage of these plants within ecosystems (Yelenik and Levine, 2011) because their high-quality litter increases soil nutrients, thereby promoting their own biomass accumulation and reproductive growth. Such increased nutrient availability may bolster their competitive ability and contribute to their successful dispersal (Patil et al., 2020; Hou et al., 2022). However, contrary to reports on other exotic plants (Meisner et al., 2012; Rothstein et al., 2004), our experiments revealed that litter inputs from *L. racemosa* actually led to a decrease in soil nutrient levels. Similar outcomes were observed for the invasive plant *Agropyron cristatum* (Christian and Wilson, 1999). This finding raises the questions of how the reduction in soil nutrients caused by *L. racemosa* litter aids the spread of this plant and what advantages are conferred by this strategy. In low-resource environments, the competitive ability of invasive plants is often influenced by their ability to enhance resource availability and tolerate low-resource conditions (Funk, 2013). Adaptations to low-resource ecosystems are frequently associated with traits related to resource acquisition or conservation (Chapin, 1980; Craine, 2009). Previous research indicates that the genetic and rhizosphere microbial characteristics of *L. racemosa* enhance its ability to acquire nutrients and tolerate low-nutrient conditions (Zhu et al., 2023; Liu et al., 2020), potentially strengthening its competitive ability in nutrient-poor environments. Therefore, we hypothesized that *L. racemosa* may employ a unique invasion strategy in which it reduces soil nutrient levels in native mangroves through low-quality litter inputs. This reduction in quality reduces the competitive advantage of native mangrove species intolerant to low-nutrient conditions but enhances the competitive advantage of *L. racemosa*, which is tolerant of low-nutrient environments (Liu et al., 2020). Similarly, Leary et al. (2006) found that litter of the invasive plant *Ulex europaeus* causes lower soil nutrient contents than that of native plants; thus, *U. europaeus* exhibits a competitive advantage over *Panicum virgatum* under nutrient-poor conditions.

The carbon content of mangroves is among the highest relative to other ecosystems (Friess et al., 2021; Macreadie et al., 2021); thus, mangroves play a crucial role in global carbon capture and storage (Lu et al., 2024). Nutrients from leaf litter decomposition are a primary source of organic matter in mangrove soils (Kristensen et al., 2008). Our single decomposition experiment and cumulative effect experiments demonstrated that the input of *L. racemosa* leaf litter decreased the soil organic carbon content. This reduction in soil organic carbon may be due to changes in microbial community structure caused by *L. racemosa* leaf litter, which enhanced microbial respiration and accelerated the conversion of soil carbon to carbon dioxide (CO_2_), leading to a decrease in organic carbon sequestration in the soil. Exotic plants, especially invasive ones, typically have high biomass and rapid growth rates, and *L. racemosa* is no exception (Li et al., 2020; Feng et al., 2022). These high-biomass, fast-growing plants return more organic carbon to the soil each year, potentially leading to increased carbon sequestration within the soil ecosystem (Cheng et al., 2006). Traditionally, such plants have been considered high carbon-sequestration species. However, in addition to plant biomass, the decomposition rates and patterns of leaf litter are also crucial factors in determining soil carbon storage. If high-biomass plants experience elevated levels of mineralization during litter decomposition, then additional carbon will be released back into the atmosphere, resulting in less organic carbon retained in the soil (Yan et al., 2018). Studies on terrestrial and wetland ecosystems indicate that the input of exotic plant litter can alter the soil microbial community structure by increasing the proportion of microbial communities responsible for mineralization (e.g., Proteobacteria), which can affect organic carbon decomposition patterns. This change can lead to increased soil respiration and CO_2_ emissions, thereby reducing soil organic carbon storage (Meisner et al., 2012; Spirito et al., 2014; Zhang et al., 2014; Yan et al., 2018). Our results indirectly support this mechanism.

## 5 Conclusion

The results of our study confirm our hypotheses that *L. racemosa* leaf litter input accelerates nutrient cycling in native mangrove forests while reducing soil nutrient contents and altering soil microbial communities. Compared with the native mangrove species (*R. stylosa*), the exotic mangrove *L. racemosa* exhibited lower leaf litter quality and faster decomposition rates. *Laguncularia racemosa* leaf litter input decreased the quality of the native mangrove soil and shifted the microbial communities toward those conducive to accelerated leaf decomposition rates and increased nutrient release rates, which accelerated nutrient cycling. Changes in the soil microbial community structure induced by *L. racemosa* leaf litter are likely responsible for the higher leaf litter decomposition rates in the invaded native mangrove forests compared with that observed for the native species because *L. racemosa* leaf litter is of lower quality. The soil nutrient content accumulated over several years under *L. racemosa* was lower than that under *R. stylosa*. This may be because nutrients released from decomposing leaf litter are absorbed by *L. racemosa* plants to support their growth and development. Furthermore, adding exotic mangrove *L. racemosa* leaf litter reduced the soil blue carbon content, which may adversely affect global climate change. The results of our study suggest that the spread of *L. racemosa* may differ from that of other exotic plants because it lowers the soil nutrient levels through lower-quality leaf litter, thereby reducing the competitive ability of native mangrove species that are intolerant to low-nutrient conditions. The enhanced competitiveness of *L. racemosa* under low-nutrient conditions likely contributed to its successful spread in the study area.

## Data Availability

The datasets presented in this study can be found in online repositories. The names of the repository/repositories and accession number(s) can be found below: https://www.ncbi.nlm.nih.gov/, PRJNA1132499.
