# Supplementary material for: Exotic mangrove Laguncularia racemosa litter input accelerates nutrient cycling in mangrove ecosystems
Source: Front Plant Sci. 2024 Oct 8;15:1463548. doi: 10.3389/fpls.2024.1463548 (PMC11493641; doi:10.3389/fpls.2024.1463548)
Supplement: Supplementary file 1 [file DataSheet1.docx]

Supplementary Materials


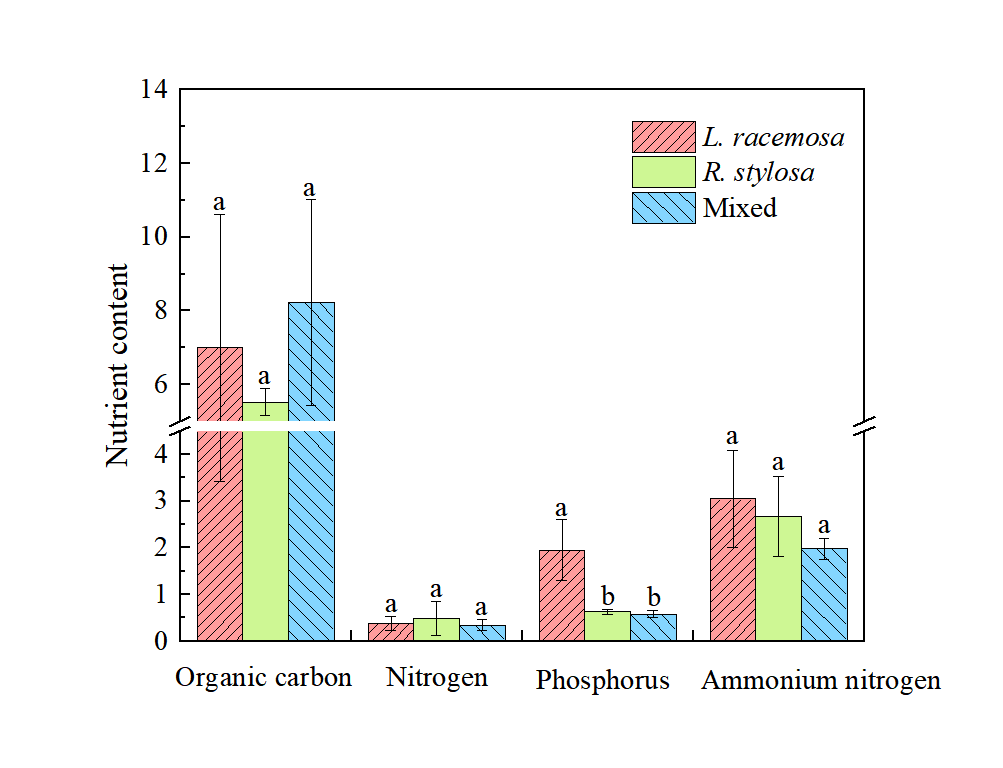


**Supplementary Figure 1.** Soil element contents of three forest types. Different lowercase letters indicate significant differences in mean soil element contents at p < 0.05. Excluding ammonium nitrogen, which is measured in milligrams per kilogram (mg/kg), the units for the other three are grams per kilogram (g/kg).

|  | **Pearson’s correlation analysis** | **C** | **N** | **P** | **Lignin** | **Cellulose** | **Lignin/C** | **Lignin/N** | **Lignin/P** | **C/N** | **C/P** | **N/P** |
| --- | --- | --- | --- | --- | --- | --- | --- | --- | --- | --- | --- | --- |
| **MRD** | Correlation coefficient | 0.356 | -0.016 | -0.075 | -0.21 | -0.01 | -0.358 | -0.054 | -0.003 | 0.111 | 0.202 | 0.104 |
|  | P | 0.068 | 0.938 | 0.711 | 0.294 | 0.959 | 0.067 | 0.79 | 0.988 | 0.583 | 0.313 | 0.607 |

**Supplementary Table 1.** Correlation between mean residue decomposition rate (MRD) and initial litter quality. Significance levels are denoted by asterisks; *p < 0.05 and **p < 0.01.

| **Nutrient element** | **Desulfobacterota** | **Bacteroidota** | **Gemmatimonadota** | **Nitrospirota** | **Spirochaetota** | **Campilobacterota** | **NB1-j** | **Zixibacteria** | **SAR324_clade** | **Patescibacteria** | **Bdellovibrionota** | **Fusobacteriota** | **FW113** |
| --- | --- | --- | --- | --- | --- | --- | --- | --- | --- | --- | --- | --- | --- |
| Organic carbon | .683* | 0.483 | -0.600 | -0.517 | 0.133 | .817** | -.700* | -0.133 | -.733* | .700* | -0.433 | .933** | 0.639 |
| Nitrogen | 0.519 | .669* | -.879** | -.669* | -0.335 | .787* | -0.477 | -0.594 | -.828** | 0.276 | 0.109 | 0.653 | .761* |
| Phosphorus | -0.167 | 0.317 | -0.450 | -0.433 | -.700* | 0.167 | 0.083 | -.833** | -0.250 | -0.500 | .800** | -0.167 | 0.146 |
| Ammonium nitrogen | .736* | 0.519 | -.870** | -0.527 | 0.042 | .695* | -0.594 | -0.469 | -.787* | 0.452 | -0.117 | 0.611 | .752* |

**Supplementary Table 2.** Correlation between the top 30 microbial phyla, based on relative abundance, and soil nutrient elements in the three forest types. *p < 0.05 and **p < 0.01.

| **MRD and Nutrient element** | Proteobacteria | Myxococcota | Chloroflexi | Bdellovibrionota | Fusobacteriota | Patescibacteria | Fibrobacterota | Ascomycota | unidentified | Basidiomycota | Mortierellomycota | Olpidiomycota |
| --- | --- | --- | --- | --- | --- | --- | --- | --- | --- | --- | --- | --- |
| MRD | -.800** | 0.017 | -0.083 | 0.033 | -0.317 | -0.233 | 0.100 | -0.083 | 0.150 | 0.000 | -.778* | 0.366 |
| Organic carbon | 0.300 | -0.483 | 0.550 | -0.533 | .750* | 0.650 | -0.283 | -0.417 | 0.617 | 0.450 | 0.335 | 0.297 |
| Nitrogen | 0.233 | -0.600 | .683* | -0.600 | 0.650 | .800** | -0.083 | -0.533 | .767* | 0.633 | 0.109 | 0.495 |
| Phosphorus | -0.067 | -.817** | 0.500 | -.900** | 0.650 | .700* | -0.300 | -.833** | .817** | .850** | -0.050 | 0.604 |
| Ammonium nitrogen | -0.167 | -0.084 | -0.435 | -0.251 | -0.134 | -0.042 | -.778* | -0.251 | -0.067 | 0.201 | 0.235 | 0.189 |

**Supplementary Table 3.** Correlation between the top 30 microbial phyla, based on relative abundance, and soil nutrient elements in the three decomposition sites.*p < 0.05 and **p < 0.01.

**Supplementary Figure 2.** Accumulative bar charts of relative abundance of (A) bacterial and (B) fungal phyla across three sites, and (C) bacterial and (D) fungal phyla after decomposition.
